# Enhancement of Electrical Characteristics and Stability of Amorphous Si-Sn-O Thin Film Transistors with SiO_x_ Passivation Layer

**DOI:** 10.3390/ma11081440

**Published:** 2018-08-15

**Authors:** Xianzhe Liu, Weijing Wu, Weifeng Chen, Honglong Ning, Xiaochen Zhang, Weijian Yuan, Mei Xiong, Xiaofeng Wang, Rihui Yao, Junbiao Peng

**Affiliations:** 1 State Key Laboratory of Luminescent Materials and Devices, South China University of Technology, Guangzhou 510640, China; msliuxianzhe@mail.scut.edu.cn (X.L.); wuwj@scut.edu.cn (W.W.); chenweifengchn@foxmail.com (W.C.); zhangxc_scut@foxmail.com (X.Z.); g18826075867@163.com (W.Y.); xiaochanglang@163.com (M.X.); psjbpeng@scut.edu.cn (J.P.); 2 Shenzhen China Star Optoelectronics Technology Co., Ltd. (CSOT), Shenzhen 518132, China; 3 Institute of Semiconductors, Chinese Academy of Science, Beijing 100083, China; wangxiaofeng@semi.ac.cn

**Keywords:** amorphous Si-Sn-O semiconductor, SiO_x_ passivation layer, density of states, stability

## Abstract

In this research, a passivated methodology was proposed for achieving good electrical characteristics for back-channel-etch (BCE) typed amorphous Si-Sn-O thin film transistors (a-STO TFTs). This methodology implied that the thermal annealing (i.e., pre-annealing) should be carried out before deposition of a SiO_x_ passivation layer. The pre-annealing played an important role in affecting device performance, which did get rid of the contamination of the lithography process. Simultaneously, the acceptor-like sub-gap density of states (DOS) of devices was extracted for further understanding the reason for improving device performance. It found that the SiO_x_ layer could reduce DOS of the device and successfully protect the device from surroundings. Finally, a-STO TFT applied with this passivated methodology could possess good electrical properties including a saturation mobility of 4.2 ± 0.2 cm^2^/V s, a low threshold voltage of 0.00 V, a large on/off current ratio of 6.94 × 10^8^, and a steep subthreshold swing of 0.23 V/decade. The threshold voltage slightly shifted under bias stresses and recovered itself to its initial state without any annealing procedure, which was attributed to the charge trapping in the bulk dielectric layers or interface. The results of this study indicate that a-STO TFT could be a robust candidate for realizing a large-size and high-resolution display.

## 1. Introduction

Amorphous metal oxide semiconductors (AMOS) are attractive candidate materials for fabricating next-generation thin film transistors (TFTs), which can be used as the driving backplanes of active matrix liquid-crystal displays (AMLCD) and active matrix organic light-emitting diode (AMOLED) displays, due to their low-temperature process, high optical transparency in the visible region, high field effective mobility, low subthreshold swing, and high on/off current ratio [1,2]. Especially, amorphous In-Ga-Zn-O (a-IGZO) has been achieved in commercial application of AMLCD or AMOLED products. With the advances in display technology, demands for large-size and high-resolution display products are increasing. The fabrication of miniaturized TFT with the back-channel-etch (BCE) technique can meet the demands of large-size and high-resolution display panels. However, the development of miniaturized AMOS TFTs is sluggish because oxide semiconductors are sensitive to weak acid etchant, causing damage to the back-channel surface of the device. Thus, exploiting an acid-resistant and stable active layer material is one potential solution for fabricating BCE typed TFTs. Various kinds of oxide semiconductors have sprung up and been widely researched in order to achieve an excellent device performance since a-IGZO was reported [3,4]. Among these oxide semiconductors, tin oxide (SnO_2_), with high chemical stability, low cost, and nontoxicity, was proposed as one of the promising candidates for fabricating BCE typed devices to enable high resolution [5,6]. However, due to polycrystalline structure and high carrier concentration of the intrinsic SnO_2_, the application of TFT backplane is limited. Recently, the doping element of silicon (Si) was successfully demonstrated to be an excellent carrier suppressor in oxide semiconductors that were used for electron injection/transport of OLED [7], the formation of Schottky contact in IGZO-TFT [8], and the active layers in oxide TFTs [9]. It is well known that the charge carriers in the oxide semiconductors can be related to the oxygen vacancy. For SnO_2_, the oxygen vacancy can be suppressed by the Si element because oxygen bond-dissociation energy of Si-O (799.6 kJ/mol) is stronger than that of Sn-O (531.8 kJ/mol), which can prevent the oxygen atoms from getting out of the SnO_2_ film during the sputtering or annealing process [10,11,12].

For integrated circuit applications of oxide TFTs, the passivation layer plays a critical role in protecting devices from surroundings. Generally, oxide semiconductors are susceptible to oxygen or water molecules in the ambient atmosphere. The adsorption/desorption dynamic of O_2_ or H_2_O molecules on the back-channel region of the device affects the properties of oxide TFT [13]. The adsorbed oxygen captures electrons from the channel layer, which results in a positive V_th_ shift in oxide TFTs ($O_{2\left(g\right)}+e^{-}↔O_{2\left(s\right)}^{-}$). The absorbed water molecules can be charged and generate excess electrons into the channel layer, which leads to a negative V_th_ shift in oxide TFTs ($H_{2}O_{\left(g\right)}↔H_{2}O_{\left(s\right)}^{+}+e^{-}$). Up until now, different species of organic and inorganic passivation layers, such as perfluoro (1-butenyl vinyl ether) polymer (CYTOP) [14], silicon nitride (SiN_x_) [15], and silicon oxide (SiO_x_) [16] have been widely studied. The diffusion barrier properties against O_2_ and H_2_O molecules in the ambient of organic passivation layers are lower than that of inorganic passivation layers on account of their high permeability of ambient gas. Although the diffusion barrier properties of SiN_x_ against O_2_ and H_2_O molecules in the ambient are better than that of SiO_x_, the generation of hydrogen concentration in SiN_x_ is higher than that of SiO_x_ during the deposition by plasma enhanced chemical vapor deposition (PECVD), which can deteriorate device performance and stability [17].

In this paper, comparison of the electrical characteristics of BCE typed a-STO TFTs, with and without thermal annealing before deposition of SiO_x_, was carried out. It demonstrated that the pre-annealing was an important step for enhancing electrical properties of the device. The reason for achieving good electrical properties of a-STO TFTs with SiO_x_ passivation was investigated in detail.

## 2. Materials and Methods

**Figure 1:**
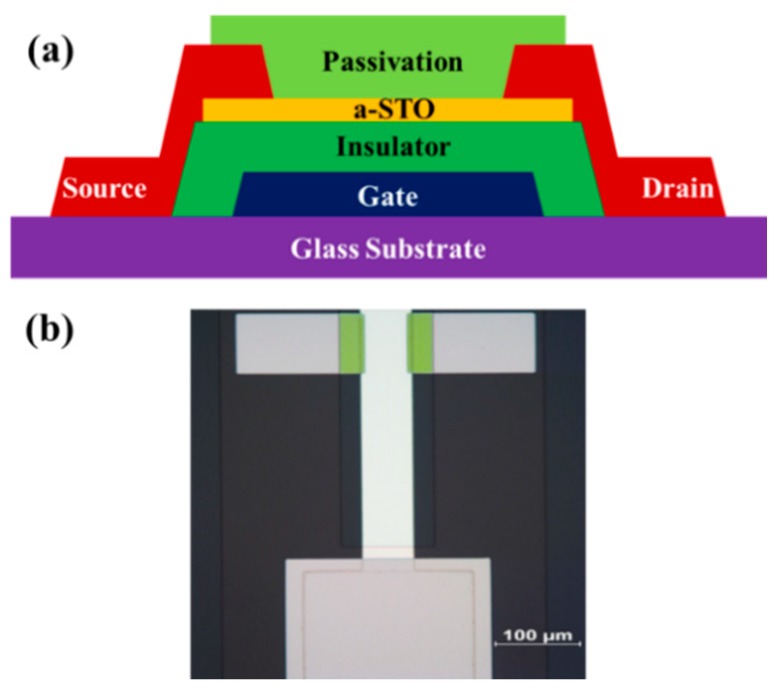
(**a**) Schematics configuration of back-channel etching type a-STO TFT. (**b**) Optical top view image of a-STO TFT. TFT channel width/length is 100 µm/50 µm.

The cross-sectional configuration schematic of a-STO TFTs with BCE structure was fabricated on glass substrates, as illustrated in Figure 1. First, a 300 nm thick Al-Nd alloy (3 wt% of Nd) was deposited on glass substrate and patterned as a gate electrode. Second, a 200 nm thick gate insulator layer of AlO_x_-Nd was formed using the anodization process. Third, an ultra-thin 5 nm a-STO film was deposited by using a ceramic target (SiO_2_:SnO_2_ = 5:95 wt%) with a power of 300 W, an argon/oxygen flow ratio of 20/2 sccm and a pressure of 2 mtorr at room temperature, and patterned by a lift-off process. Fourth, a 200 nm Mo film used for the Source/Drain (S/D) electrodes was sputtered at room temperature and the channel width/length (100/50 μm) was defined using the wet etch process. Fifth, the passivation-free Device A was fabricated. Finally, two passivation process approaches of 300 nm SiO_x_ deposited by PECVD at 300 °C were conducted, respectively. A layer of SiO_x_ film was directly deposited on the top of Device A, which was called Device B. The channel region of Device A was covered by a SiO_x_ layer after being annealed at 350 °C in air ambient for 0.5 h, which resulted in the formation of Device C (See Figure S1).

Metal electrodes were prepared by direct current (DC) magnetron sputtering and a-STO films were deposited by radio frequency (RF) Magnetron sputtering (Kurt J. Lesker, Jefferson Hills, MA, USA). Morphology and roughness of a-STO film deposited on the glass substrate were obtained by atomic force microscope (AFM, Multimode 8, Bruker, Karlsruhe, Germany) measurement. The density and thickness were analyzed by X-ray reflectivity (XRR, EMPYREAN, PANalytical, Almelo, The Netherlands). The oxygen vacancies of the channel layer were measured by X-ray photoelectron spectroscopy (XPS, Escalab 250XI, Thermo Scientific, Waltham, MA, USA). The electrical characteristics and stability of device under gate bias stress were measured in air ambient by an Agilent 4155C semiconductor parameter analyzer (Agilent, Santa Clara, CA, USA). The applied positive bias stress (PBS) and negative bias stress (NBS) were as follows: V_GS_ = ±20 V and V_DS_ = 0 V, which was applied for 3600 s. The transfer characteristic curves of the device were tested when the gate biases were interrupted at fixed times. V_th_ was extracted from the linear extrapolation of the plot of the square root of the drain current with gate voltage. And the recovery of V_th_ was also investigated in the dark after bias stress for 3600 s. The transfer characteristic curves of TFT were measured after 1200 s intervals. Capacitance–voltage (C–V) curves of a-STO TFTs were measured by a KEYSIGHT E4990A Impedance Analyzer (Keysight Technologies Inc., Santa Rosa, CA, USA) with a fixed frequency of 10 k Hz. The 2D device simulator ATLAS (Silvaco Inc., Santa Clara, CA, USA) was used for device simulation.

## 3. Results and Discussion

**Figure 2:**
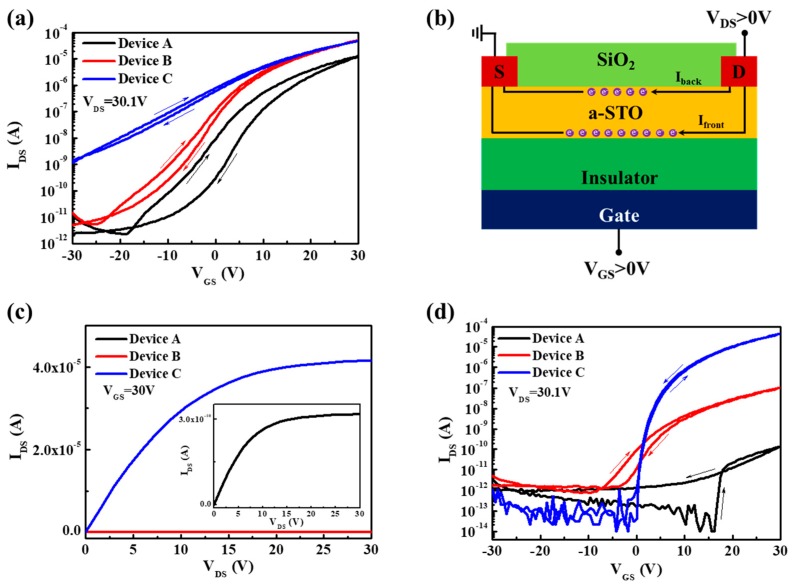
(**a**) The transfer characteristic curves of as-deposited a-STO TFTs when V_DS_ was fixed at 30.1 V; (**b**) Schematic of current paths of as-deposited a-STO TFT; (**c**) The drain current of a-STO TFTs annealed at 450 °C for 0.5 h in argon ambient when V_GS_ was fixed at 30 V; (**d**) The transfer characteristic curves of a-STO TFTs annealed at 450 °C for 0.5 h in argon ambient when V_DS_ was fixed at 30.1 V.

Figure 2a shows the representative transfer characteristic curves (I_DS_–V_GS_) of as-deposited a-STO TFTs. There were many intrinsic defects at the semiconductor/insulator interface or in the a-STO active layer film for as-deposited Device A, which led to the generation of poor electrical performance, such as a large subthreshold swing (SS) and a large hysteresis. An evident increase of OFF current (I_off_) and negative shift of turn-on voltage (V_on_) were found after the channel region of the device was covered by a SiO_x_ passivation layer. Generally, the channel current (I_front_) of the device cannot be affected by a passivation layer [18]. Thus, the increase of I_off_ in the passivated device was attributed to the byproduct hydrogen during the deposition of SiO_x_. Because neutral H^0^ could migrate in the channel layer and further react with O^2−^ to form OH^−^ and generate excessive electrons ($H^{0}+O^{2-}\to {\mathrm{OH}}^{-}+e^{-}$), which would result in the formation of back-channel current (I_back_), as shown in Figure 2b [19].

To obtain good performance of a-STO TFTs, an additional thermal post-annealing in argon ambient for devices was inevitably carried out. The I–V curves of both Device A (~10^−10^ A) and Device B (~10^−8^ A) were approximate to a horizontal line compared with that of device C (~10^−5^ A), as illustrated in Figure 2c. Figure 2d exhibits the transfer characteristic curves of devices with thermal post-annealing. The transfer characteristics of Device A and Device B were not improved by post-annealing. The channel layer of Device A was terribly transformed into an insulator-like layer. For the transfer curve of Device B, the hysteresis unexpectedly was still maintained and the reduction of ON current (I_on_) also occurred. Fortunately, a good device performance was successfully achieved in Device C. The field-effect mobility in the saturation region of the device and the subthreshold swing (SS) were extracted by using the following equations [1]:

(1) $$I_{\mathrm{DS}}=\frac{{W\mu }_{\mathrm{sat}}C_{i}}{2L}{\left(V_{\mathrm{GS}}-V_{\mathrm{th}}\right)}^{2}$$

(2) $$\mathrm{SS}={\left(\frac{\mathrm{dlog}\left(I_{\mathrm{DS}}\right)}{{\mathrm{dV}}_{\mathrm{GS}}}\right)}^{-1}$$

where I_DS_ is the drain current, V_DS_ is the drain voltage, V_GS_ is the gate voltage, V_th_ is the threshold voltage, W/L is the channel width/length, and C_i_ is the gate capacitance per unit area of the insulator layer, respectively. The electrical parameters of devices with the post-annealing process, including saturation mobility (μ_sat_), turn-on voltage (V_on_), on/off current ratio (I_on_/I_off_), and subthreshold swing (SS) were listed in Table 1. A large μ_sat_ of 4.2 ± 0.2 cm^2^/V s, a low V_on_ of 0.00 V, a large I_on_/I_off_ of 6.94 × 10^8^, and a low SS of 0.23 V/decade were obtained in Device C.

**Figure 3:**
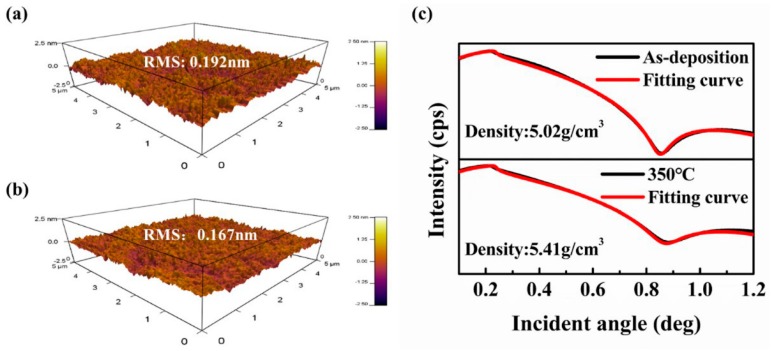
AFM images (5 × 5 μm) of 5 nm a-STO films: (**a**) as-deposition and (**b**) 350 °C. (**c**) The XRR curves of 5 nm a-STO films.

The results of device performance implied that the pre-annealing played an important role in affecting the electrical properties of passivated device. An in-depth investigation of the thermal pre-annealing for Device A was performed. Figure 3 shows AFM images and XRR curves of 5 nm a-STO films. After a-STO film was annealed at 350 °C in air ambient for 0.5 h, the roughness reduced from 0.192 nm to 0.167 nm, while the density of a-STO film increased from 5.02 g/cm^3^ to 5.41 g/cm^3^. The thermal annealing process could facilitate the atoms’ rearrangement and structural relaxation to reduce internal defects and improve the quality of film.

**Figure 4:**
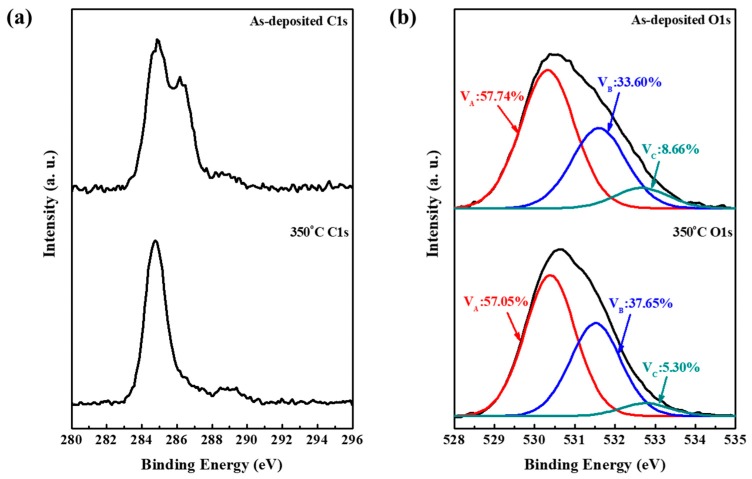
Comparison of XPS spectra in the channel region of Device A with/without thermal annealing process of 350 °C in air ambient: (**a**) C1s spectra and (**b**) O1s spectrum.

Currently, oxygen vacancies are closely related to the field-effect mobility and threshold voltage of metal-oxide-semiconductor devices, according to the equation: $O_{O}^{X}=\frac{1}{2}O_{2\left(g\right)}+V_{O}^{**}+2e^{-}$ [20]. The XPS analysis of the channel region was implemented, as displayed in Figure 4. All binding energies were corrected by referencing to the C1s peak (centered at 284.8 eV). From the C1s spectra in Figure 4a, it clearly revealed that the photoresist was not thoroughly eliminated in the channel region of as-deposited devices. And the contamination was dramatically eliminated in the channel region after being annealed at 350 °C in air ambient. The O1s peak of a-STO channel layer could be de-convoluted into three principal sub-peaks via using a Gaussian-Lorentzian profile, which centered at 530.5 ± 0.3 eV (peak A), 531.8 ± 0.2 eV (peak B), and 532.7 ± 0.1 eV (peak C), respectively. The lowest binding energy located at 530.5 ± 0.3 eV was associated with oxygen-lattice bonds (Sn-O and Si-O) in a-STO compound system. The middle binding energy, centered at 531.8 ± 0.2 eV, was assigned to the oxygen vacancies. The highest binding energy positioned at 532.7 ± 0.1 eV was usually attributed to the presence of loosely bound oxygen on species, with a surface of the a-STO film such as -CO_3_, -OH or adsorbed O_2_. After the unpassivated device was annealed at 350 °C, a distinct increased area proportion of V_B_ (B/(A + B + C)), from 33.60% to 37.64%, and an obvious decreased proportion of V_C_ (C/(A + B + C)), from 8.66% to 5.30%, were found. These results indicated that the pre-annealing could not only effectively remove the contamination but also facilitate the increase of carrier concentration.

**Figure 5:**
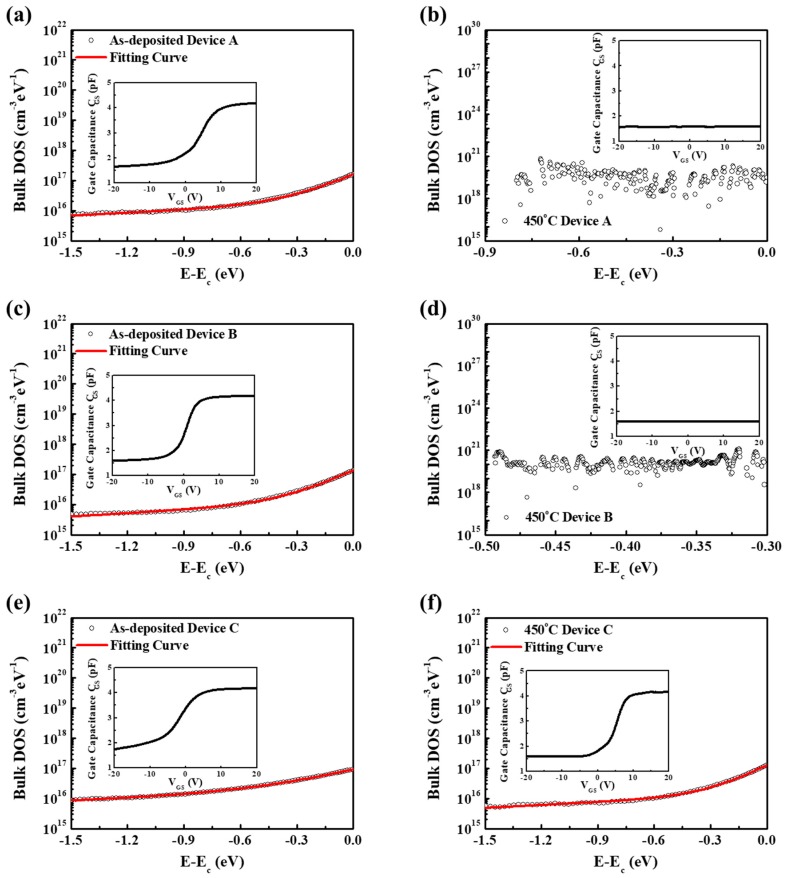
Extracted DOS of a-STO TFTs as function of E − E_c_: (**a**) as-deposited Device A, (**b**) 450 °C Device A, (**c**) as-deposited Device B, (**d**) 450 °C Device B, (**e**) as-deposited Device C and (**f**) 450 °C Device C. Inset is C-V curve of the corresponding a-STO TFT at 10 k Hz.

In AMOS devices, the sub-gap density of states (DOS) is an important parameter closely related to the mobility, operation voltage, and subthreshold swing of TFTs. To gain an insight into the reason for a series of changes in device performance, the acceptor-like DOS of a-STO TFTs was extracted by a low-frequency capacitance-voltage (C-V) characteristic [21]. The results of C-V characteristics and DOS of a-STO TFTs were exhibited in Figure 5. The DOS of devices might be divided into two parts: deep states and tail states, which could be approximately represented by the superposition of the exponential tail states and exponential deep states:

(3) $$N_{t}\left(E\right)=N_{\mathrm{DA}}\exp\left(\frac{E-E_{c}}{E_{\mathrm{DA}}}\right)+N_{\mathrm{TA}}\exp\left(\frac{E-E_{c}}{E_{\mathrm{TA}}}\right)$$

where N_DA_, N_TA_, E_DA_, E_TA_, E, and E_c_ is the density of deep states, the density of tail states, the energy of deep states, the energy of tail states, the state energy, and the conduction band minimum, respectively. The N_DA_/N_TA_ is extracted by extrapolating the deep/tail states to E = E_c_, while E_DA_/E_TA_ is extracted from the slope of log(N_t_) versus (E − E_c_) for the deep/tail states. The fitting results of DOS of a-STO TFTs were shown in Table 2. The density of deep/tail states (2.02 × 10^18^/4.61 × 10^20^ cm^−3^ eV^−1^) of Device A annealed at 350 °C in air ambient was obtained (See Figure S2). The passivation layer could effectively degrade DOS of device. Quantities of defects were generated in the Device A and Device B after they were annealed at 450 °C in argon ambient, which led to the deterioration of device performance. For Device C, the density of deep states decreased from 1.97 × 10^16^ cm^−3^ eV^−1^ to 1.44 × 10^16^ cm^−3^ eV^−1^ after thermal post-annealing, while the density of tail states increased from 0.73 × 10^17^ cm^−3^ eV^−1^ to 1.13 × 10^17^ cm^−3^ eV^−1^. The reason for the decrease of density of deep states was attributed to the reduction of excess oxygen in a-STO film [22]. Due to the existence of nonstoichiometric SiO_x_, excess oxygen in the bulk a-STO film was attracted to the SiO_x_/a-STO interface after post-annealing (See Figure S3). The increase of density of tail states might be associated with the elimination of hydrogen (${\mathrm{OH}}^{-}\to H^{o}+O^{-}$) because the $O^{-}$ ion can capture electrons to form stable $O^{2-}$.

**Figure 6:**
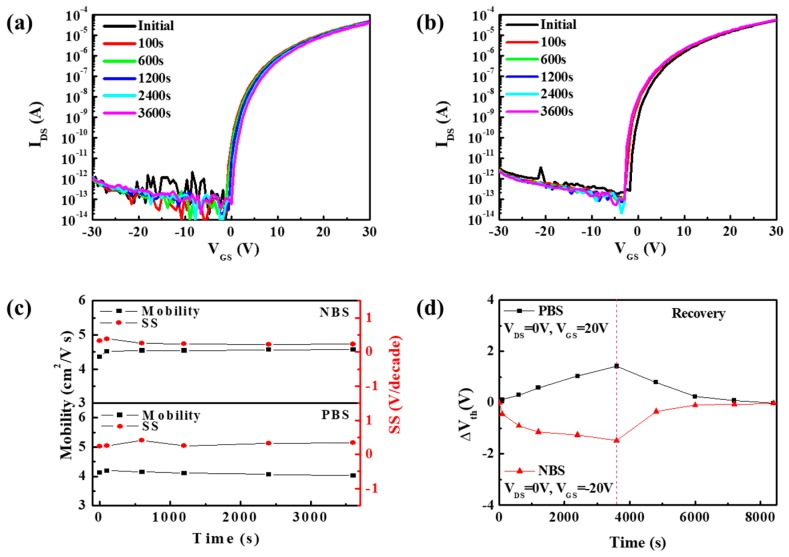
The evolution of transfer characteristic curves of device C annealed at 450 °C in vacuum argon ambient under (**a**) dark PBS condition and (**b**) dark NBS condition. (**c**) The change of mobility and SS of device C as a function of stress time under PBS condition and NBS condition. (**d**) Variation in the V_th_ value as a function of stress time and recovery time. The pink dash line is at the position of 3600 s.

**Figure 7:**
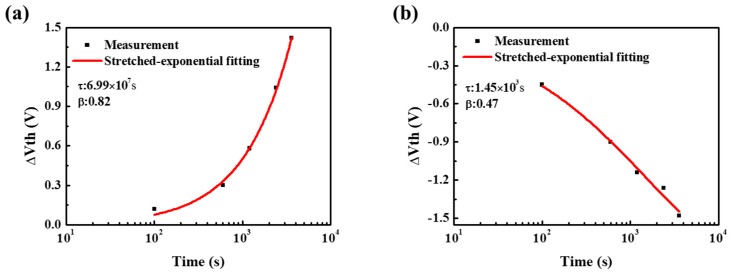
Time evolution of ΔV_th_ under different bias stresses: (**a**) PBS and (**b**) NBS. The measured data was well fitted with a stretched-exponential equation in both phases.

The electrical stability is a very important parameter for AMOS-TFTs backplane in display. The investigation of PBS and NBS electrical instabilities of Device C was performed, as illustrated in Figure 6a,b. The threshold voltage shift (ΔV_th_) under stress phase was 1.42 V (PBS) and −1.48 V (NBS), respectively. After both bias stresses were applied, the V_th_ of device could spontaneously recover to its initial state after a period of relaxation of 4800 s. It is well known that there are two major mechanisms causing V_th_ instability: (1) defect creation in the channel and (2) charge trapping in the gate insulator and/or at the channel/insulator interface [23]. The negligible variation of mobility and SS (Figure 6c) and the recovery of V_th_ without any annealing procedure (Figure 6d) after relaxation indicated that the charge trapping in the bulk dielectric layers or interface was the dominant mechanism responsible for V_th_ instability. Thus, the charge trapping model could be reflected in the stress time dependence of ΔV_th_, well-fitted with the stretched-exponential equation [24]:

(4) $$\Delta V_{\mathrm{th}}=\Delta V_{\mathrm{th}0}\left\{1-\exp\left[-{\left(\frac{t}{\tau }\right)}^{\beta }\right]\right\}$$

where ΔV_th0_ is the ΔV_th_ at infinite time, t is the stress time, τ is the characteristic time constant for trapping, and β is the stretched-exponential exponent, respectively. Figure 7 shows the fitting curves of time dependence of ΔV_th_ under PBS and NBS conditions. The characteristic time constant τ was 6.99 × 10^7^ s (PBS) and 1.45 × 10^3^ s (NBS) while stretched-exponential exponent β was 0.82 (PBS) and 0.47 (NBS), respectively.

## 4. Conclusions

In summary, a good performance and stability of a-STO TFTs with BCE-typed structure was achieved by covering a SiO_x_ passivation layer. The thermal pre-annealing was an important factor for influencing device performance. It could effectively remove the contamination in the back-channel region of the device. The threshold voltage shift (ΔV_th_) of a-STO TFTs was 1.42 V (PBS) and −1.48 V (NBS) with slight variation of mobility and SS under PBS and NBS conditions, respectively. The time-evolution ΔV_th_ of a-STO TFT under different stresses were well fitted by the stretched-exponential equation. After applied bias stress, the V_th_ of the device could spontaneously recover to its initial state after a period of relaxation of 4800 s. The reason for the instabilities of a-STO TFTs was attributed to the charge trapping in the bulk dielectric layers or interface. The results of this study show that a good performance and stability of a-STO TFTs represent good potential for display applications.

## Figures and Tables

**Table 1:** Electrical parameters of a-STO TFTs with post-annealing in argon ambient.

Sample	μ_sat_ (cm^2^/V s)	V_on_ (V)	I_on_/I_off_	SS (V/decade)
Device A	-	-	-	-
Device B	-	−9.55	1.37 × 10^5^	3.39
Device C	4.2 ± 0.2	0.00	6.94 × 10^8^	0.23

μ_sat_: saturation mobility, V_on_: turn-on voltage, I_on_/I_off_: on/off current ratio, SS: subthreshold swing.

**Table 2:** Comparison of DOS parameters of a-STO TFTs.

Samples	N_DA_ (cm^−3^ eV^−1^)	E_DA_ (eV)	N_TA_ (cm^−3^ eV^−1^)	E_TA_ (eV)
as-deposited Device A	1.96 × 10^16^	1.41	1.41 × 10^17^	0.16
450 °C Device A	-	-	-	-
as-deposited Device B	1.40 × 10^16^	1.18	1.20 × 10^17^	0.15
450 °C Device B	-	-	-	-
as-deposited Device C	1.97 × 10^16^	1.73	0.73 × 10^17^	0.26
450 °C Device C	1.44 × 10^16^	1.38	1.13 × 10^17^	0.13

## Supplementary Materials

The following are available online at http://www.mdpi.com/1996-1944/11/8/1440/s1, Figure S1: The fabricated three samples: Device A, Device B and Device C, respectively. Figure S2: (**a**) The transfer characteristic curve of Device A annealed at 350 °C in air ambient. (**b**) DOS extraction of corresponding Device A. Figure S3: The cross-sectional image and EDS scan line data of the SiO_x_/a-STO interface in Device C with post-annealing process. Method: The procedure for the proposed extraction method is described as follows. Table S1: Change in parameters including Vth, μ_sat_ and SS of a TFT as a function of stress time for different bias stress conditions: (**a**) Positive bias stress and (**b**) Negative bias stress, respectively.
