# Supplementary material for: Enhancement of Electrical Characteristics and Stability of Amorphous Si-Sn-O Thin Film Transistors with SiOx Passivation Layer
Source: Materials (Basel). 2018 Aug 15;11(8):1440. doi: 10.3390/ma11081440 (PMC6119873; doi:10.3390/ma11081440)
Supplement: Supplementary file 1 [file materials-11-01440-s001.pdf]

Enhancement of Electrical Characteristics and Stability of Amorphous Si-Sn-O Thin Film Transistors with SiO_x Passivation Layer

Xianzhe Liu [1], Weijing Wu [1], Weifeng Chen [1], Honglong Ning [1,*], Xiaochen Zhang [1], Weijian Yuan [1], Mei Xiong [1,2], Xiaofeng Wang [3], Rihui Yao [1,*] and Junbiao Peng [1]

1 State Key Laboratory of Luminescent Materials and Devices, South China University of Technology, Guangzhou 510640, China; msliuxianzhe@mail.scut.edu.cn (X.L.); wuwj@scut.edu.cn (W.W.); chenweifengchn@foxmail.com (W.C.); zhangxc_scut @foxmail.com (X.Z.); g18826075867@163.com (W.Y.); xiaochanglang@163.com (M.X.); psjbpeng@scut.edu.cn (J.P.)

2 Shenzhen China Star Optoelectronics Technology Co., Ltd. (CSOT), Shenzhen 518132, China

3 Institute of Semiconductors, Chinese Academy of Science, Beijing 100083, China; wangxiaofeng@semi.ac.cn

* Correspondence: ninghl@scut.edu.cn (H.N.); yaorihui@scut.edu.cn (R.Y.); Tel.: +86-20-8711-0606 (H.N.); +86-20-8711-4346 (R.Y.)

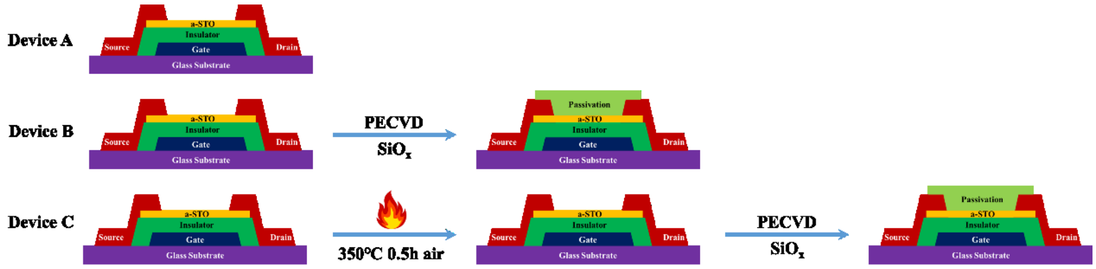

Figure S1. The fabricated three samples: Device A, Device B and Device C, respectively.

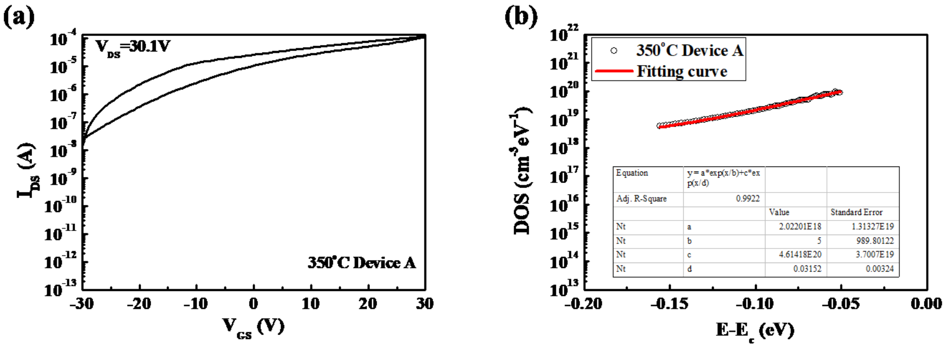

Figure S2. (**a**) The transfer characteristic curve of Device A annealed at 350 ℃ in air ambient. (**b**) DOS extraction of corresponding Device A.

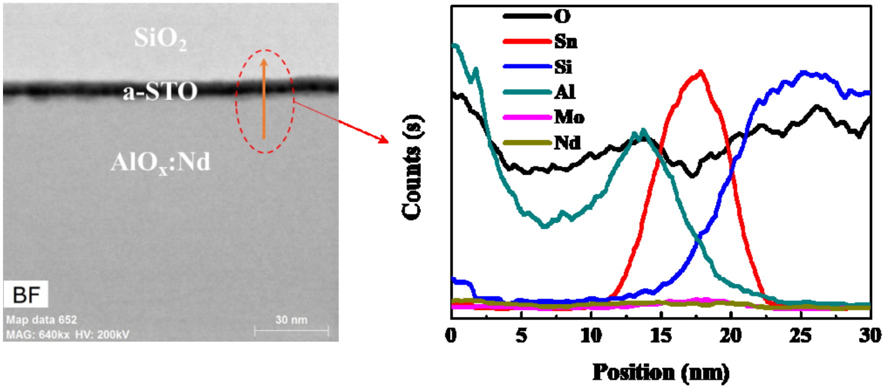

Figure S3. The cross-sectional image and EDS scan line data of the SiO_x/a-STO interface in Device C with post-annealing process.

The procedure for the proposed extraction method is described as follows:

$$\psi_s = \int_{V_{fb}}^{V_{gs}} \left(1 - \frac{C_g(V'_{gs})}{C_{ox}}\right) dV'_{gs} \quad (1)$$

$$\rho(\psi_s) = -\frac{C_g(V_{gs}) \int_{V_{fb}}^{V_{gs}} C_g(V'_{gs}) dV'_{gs}}{\varepsilon_s\left(1 - \frac{C_g(V_{gs})}{C_{ox}}\right)} \quad (2)$$

$$N_t(E_{F0} + q\psi_s) = -\frac{1}{q^2}\frac{\rho(\psi_s+\Delta\psi_s)-\rho(\psi_s)}{\Delta\psi_s} - \frac{n_0}{qV_t} exp\left(\frac{\psi_s}{V_t}\right) \quad (3)$$

Where ψ_s is the surface potential, V_{fb} is the flat band voltage, V_{gs} is the gate voltage, C_g is gate capacitance, C_{ox} is the gate oxide capacitance per unit area, $\rho(\psi_s)$ is the surface charge concentration, E_{F0} is the bulk Fermi level of the active layer and $N_t(E_{F0} + q\psi_s)$ is density of trap states with respect to some energy level ($E = E_{F0} + q\psi_s$), respectively.

Firstly, Ψ_s in terms of V_{gs} is calculated from the C_g-V_{gs} characteristics of TFTs by (1). Secondly, the surface charge concentration $\varrho(\Psi_s)$ can be obtained from the C_g-V_{gs} characteristics of TFTs by (2). Finally, the density of states $N_t(E)$ with respect to some energy level ($E = E_{F0} + q\Psi_s$) can be extracted by (3). As seen above, the proposed extraction method of DOS has the advantages of analyticity and simplicity.

Table S1. Change in parameters including V_{th}, μ_{sat} and SS of a TFT as a function of stress time for different bias stress conditions: (a) Positive bias stress and (b) Negative bias stress, respectively.

(a) Positive bias stress condition

**Time (s)**	**0**	**100**	**600**	**1200**	**2400**	**3600**
V_{th} (V)	10.71	10.83	11.01	11.29	11.75	12.13
μ_{sat} (cm^2/V s)	4.13	4.19	4.15	4.11	4.06	4.02
SS (V/decade)	0.23	0.25	0.41	0.25	0.33	0.34

(b) Negative bias stress condition.

**Time (s)**	**0**	**100**	**600**	**1200**	**2400**	**3600**
V_{th} (V)	9.78	9.33	8.88	8.64	8.52	8.30
μ_{sat} (cm^2/V s)	4.36	4.52	4.54	4.54	4.56	4.57
SS (V/decade)	0.33	0.38	0.26	0.23	0.22	0.23
